# Midgestational Uterine Rupture With Spontaneous Bladder Disruption due to Placenta Percreta: A Case Report

**DOI:** 10.1155/crog/5281510

**Published:** 2026-02-23

**Authors:** Mahdieh Mottaghi, Leila Pourali, Shahrzad Bahadorian, Laya Shirinzadeh, Atiyeh Vatanchi

**Affiliations:** ^1^ Clinical Research Development Unit, Ghaem Hospital, Mashhad University of Medical Sciences, Mashhad, Iran, mums.ac.ir; ^2^ Supporting the Family and the Youth of Population Research Core, Department of Obstetrics and Gynecology, Faculty of Medicine, Mashhad University of Medical Sciences, Mashhad, Iran, mums.ac.ir; ^3^ Department of Pathology, Faculty of Medicine, Mashhad University of Medical Sciences, Mashhad, Iran, mums.ac.ir

**Keywords:** bladder disruption, placenta percreta, pregnancy, uterine rupture

## Abstract

**Introduction:**

Uterine rupture due to placenta percreta in midgestation is rare, particularly when associated with spontaneous bladder disruption.

**Case Presentation:**

A 36‐year‐old gravida 9, para 5 woman presented at 20 weeks of gestation with complaints of vaginal bleeding triggered by urination and walking. Ultrasonography performed at 17 + 5 weeks′ gestation indicated placenta accreta spectrum with possible bladder invasion. On admission, the initial evaluation revealed stable vital signs, a closed cervix with no active bleeding, and a reactive nonstress test, and the patient was kept under observation. She later developed abrupt hypogastric pain accompanied by gross hematuria. Physical examination revealed stable vital signs, vaginal bleeding, and uterine contractions. Emergent laparotomy revealed rupture of the anterior uterine wall at the site of the previous cesarean scar, with extrusion of a nonviable fetus into the abdominal cavity and spontaneous bladder disruption due to extensive placental invasion through the uterine serosa into the bladder. Hemorrhagic adnexal cysts were incidentally identified. A subtotal hysterectomy, left salpingo–oophorectomy, and bladder repair were performed. The patient recovered well; however, during the 2‐year follow‐up, she reported symptoms of overactive bladder.

**Conclusion:**

When placenta percreta is suspected antenatally, the rare possibility of uterine rupture and risk of spontaneous bladder rupture must be kept in mind.

## 1. Introduction

Uterine rupture refers to the complete disruption of all uterine layers, including the myometrium and serosa [1]. This condition is primarily associated with prior surgical scarring of the uterus [2]. However, it can also rarely occur due to placenta percreta, with an incidence of approximately 1 in 5000 pregnant women [3].

Abnormal placentation or placenta accreta spectrum (PAS) disorders are classified based on the depth of placental invasion into three categories: placenta accreta, increta, and percreta [4]. The prevalence of PAS disorders has increased due to rising cesarean section (CS) rates [4]. Previous studies have reported that PAS can lead to uterine rupture in the late second or third trimester of pregnancy [5]. However, its occurrence in midgestation is rare and often results in catastrophic outcomes. Furthermore, what distinguishes this case from prior reports is the concurrent rupture of the uterus and bladder due to placenta percreta.

Herein, we describe a multigravida woman who experienced uterine rupture with bladder rupture during midpregnancy due to placenta percreta.

## 2. Case Presentation

**Figure 1:**
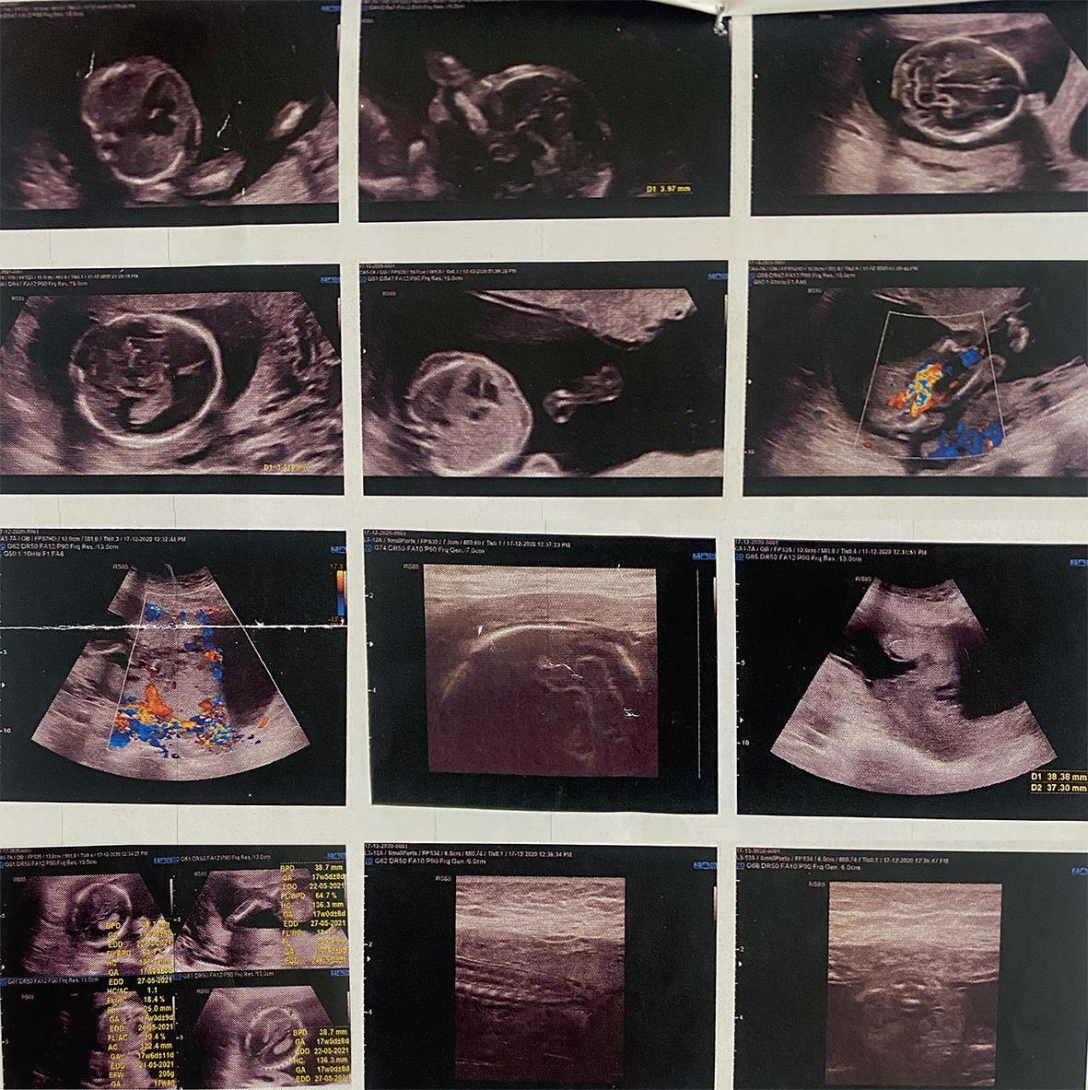
Ultrasound images obtained at 17 weeks and 5 days of gestation demonstrating anterior placenta previa with features of placenta accreta spectrum and suspected placental invasion of the bladder wall on color Doppler imaging.

A 36‐year‐old Afghan pregnant woman, gravida 9 para 5 (including four live births and one fetal death due to anencephaly), at 20 weeks′ gestation, presented with vaginal bleeding for 6 h prior to presentation, associated with micturition and walking. The patient did not report nausea, vomiting, or abdominal pain. The pregnancy was conceived naturally. Her past obstetric history included three prior CSs; the most recent was performed 3 years and 6 months before presentation. Additionally, she had a medical history of hypothyroidism and was on levothyroxine 25 *μ*g daily. She denied any history of abdominal trauma, smoking, addiction, or alcohol consumption. Her body mass index (BMI) was 24.9 kg/m^2^. An ultrasound performed at 17 weeks and 5 days of gestation (determined by fetal biparietal diameter) revealed an anterior placenta previa with features of placenta percreta and suspected bladder wall invasion (Figure 1).

Upon admission, gestational age was estimated at 20 weeks based on first‐trimester ultrasonography, as the last menstrual period (LMP) was uncertain. The patient was conscious, with a heart rate of 92 beats/min and a blood pressure of 105/65 mm Hg. The uterine fundus was consistent with her gestational age. Speculum examination showed a closed external cervical ostium with no active vaginal bleeding. A nonstress test (NST) was reactive without any significant patterns. Laboratory tests showed hemoglobin of 11.8 g/dL, white blood cells of 8.1 × 10^3^/*μ*L, and normal platelet and coagulation profiles. All other laboratory tests were also within normal limits. Abdominal ultrasonography indicated a viable intrauterine fetus with normal fetal heart rate (FHR) and activity. The placenta was anteriorly located, the fetus was in cephalic presentation, and the amniotic fluid volume was within normal limits, with an amniotic fluid index (AFI) of 14 cm. The patient was kept under observation.

After 6 h, the patient developed abrupt hypogastric abdominal pain that was continuous and worsened intermittently. On physical examination, the patient had a blood pressure of 110/62 mm Hg and a heart rate of 81 beats/min. Uterine contractions were present, and speculum examination revealed approximately 200 mL of vaginal bleeding. Cardiotocography (CTG) indicated sudden fetal distress, with the absence of fetal heart activity. An ongoing hematuria was noted upon catheterization of the bladder. Informed consent for emergency exploratory laparotomy and hysterectomy was obtained, and the patient was transferred to the operation room.

During the emergency laparotomy, a massive hemoperitoneum of blood and clots was identified and evacuated. The anterior uterine wall had completely ruptured at the site of the previous CS scar. A female 309‐g fetus, deceased and enclosed in membranes, had extruded through the rupture into the abdominal cavity. The placenta had penetrated the uterine serosa and extended into the urinary bladder. Uterine rupture resulted in subsequent bladder rupture, with large cystotomies in the dome of the bladder. The patient underwent a subtotal hysterectomy and left salpingo–oophorectomy due to simultaneous rupture of hemorrhagic adnexal cysts. The involved bladder tissue was excised, and the lacerations were repaired with three layers of 2‐0 absorbable sutures. Methylene blue was instilled into the bladder to confirm the integrity of the repair.

By the end of the surgery, the patient received 5 units of packed red blood cells, 4 units of fresh frozen plasma, and 7 units of platelets. The estimated total blood loss was approximately 4000 mL.

Postoperatively, the patient was admitted to the intensive care unit for 2 days and then transferred to the general ward. The surgical drain output was within the normal range (<100 cc/24 h), and the drain was subsequently removed.

On the fourth postoperative day, the patient was discharged with satisfactory recovery. She was prescribed tolterodine 2 mg twice daily and subcutaneous enoxaparin 40 mg daily for 10 days. A urinary catheter remained in place for 2 weeks after discharge.

On pathological evaluation, the uterus measured 10 × 9 × 4.5 cm, with a complete anterior rupture of 7 cm. Histopathological assessment of the uterine wall revealed villous invasion to the serosal side of the uterine parametrium. Furthermore, a section of the bladder measuring 3.5 × 2.5 × 0.7 cm was involved by placental villi, consistent with placenta percreta (Figure 2).

Figure 2(a) Bladder wall is lined with transitional epithelium, and muscle fibers are seen beneath the epithelium, (b) one placental villous (black arrow) invading the bladder wall, (c) placenta, villi (bottom right) and necrotic decidua (upper left), (d) placental villi (black arrow) invading myometrium with exaggerated placental site reaction (red arrow) (H&E, (a) 100×, (b) 100×, (c) 40×, and (d) 400×).

**Figure:**
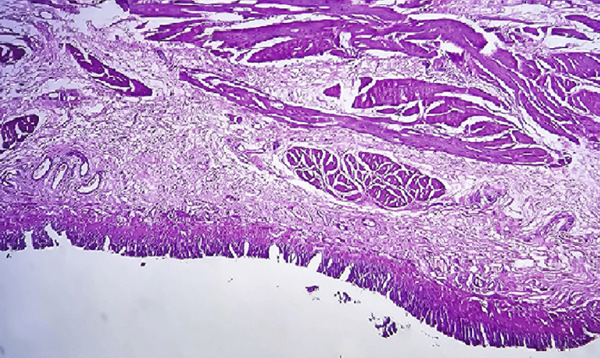
(a)

**Figure:**
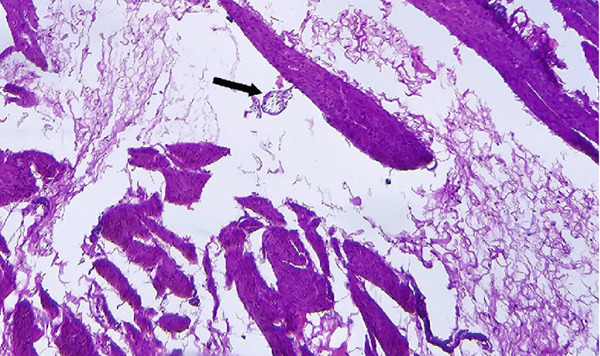
(b)

**Figure:**
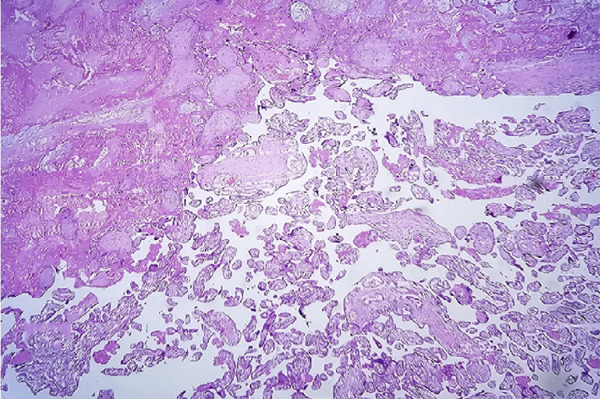
(c)

**Figure:**
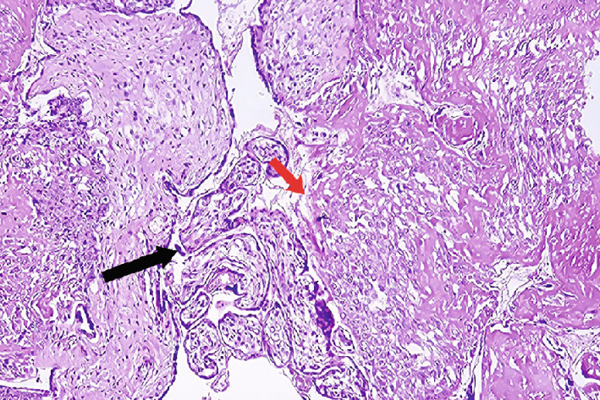
(d)

Two years postoperatively, the patient was advised to visit the urogynecology clinic for further evaluation of her urological complications. A subjective assessment of urinary symptoms was conducted using the incontinence questionnaire‐female lower urinary tract symptom (ICIQ‐FLUTS) questionnaire. Symptom frequency was scored on a scale of 0 (*never*) to 4 (*all of the time*), and symptom bother on a scale of 0 (*not at all*) to 10 (*a great deal*) (Table 1). The patient passed urine 7–8 times during the day (normal: 1–6 times) with a bother score of 6. Most of the time, the patient had urgency with a corresponding bother score of 6, followed by urge incontinence with a high bother score of 9. Urge incontinence occurred once per day, with a notable bother score of 9. Additionally, most of the time, the patient experienced stress incontinence, with a bother score of 9.

**Table 1:** Subjective assessment of urinary symptoms using the ICIQ‐FLUTS (incontinence questionnaire‐female lower urinary tract symptom) questionnaire.

Bother score(0–10)	Frequency score(0–4)	Lower urinary tract symptoms	Urinary symptoms subscale
Filling	Nocturia	0	0
	Urgency	3	6
	Bladder pain	1	2
	Frequency	1	6

Voiding	Hesitancy	0	0
	Straining	0	0
	Intermittency	0	0

Incontinence	Urge incontinence	3	9
	Urge incontinence frequency	2	9
	Stress incontinence	3	9
	Unexplained urinary incontinence	0	0
	Enuresis	0	0

*Note:* Frequency was scored on an ordinal scale (0–4), as a score of 0 means “not at all” and a score of 4 “all the time”. For daytime frequency, Score 0 shows “1–6 times” and Score 4 “≥ 13 times”; for nocturia, a score of 0 means “0 times” and Score 4 “≥ 4 times”; and for urge incontinence, Frequency Score 0 means “not at all” and Score 4 “Several times a day”. All symptoms were scored on a bother scale ranging from 0 (*not at all*) to 10 (*a great deal*).

She was trained on lifestyle modification and received medical treatment with anticholinergic medications, which led to a significant improvement in her symptoms.

## 3. Discussion

Rupture of the uterus, similar to the rupture of any internal organ, can be life‐threatening [6]. Focal ruptures of the gravid uterus can be challenging to detect by ultrasound. Additionally, the presentations of uterine rupture are often nonspecific, which further complicates diagnosis [7]. Physicians must differentiate uterine rupture from other conditions, such as the rupture of adnexal cysts, appendicitis, and renal stones [8]. This condition is associated with prior uterine surgeries, labor induction, advanced maternal age, and abnormal placentation including PAS disorders [3–5].

The uterine rupture induced by placenta percreta in our patient during midgestation can be attributed to the multiple risk factors; including high parity, advanced maternal age, multiple prior CSs, and abnormal placentation.

In addition to cesarean delivery, other uterine surgical procedures, particularly those involving deep myometrial disruption such as interstitial wedge resection [9], myomectomy, and adenomyomectomy, have been identified as significant risk factors for uterine rupture, with adenomyomectomy reported as one of the most hazardous due to extensive myometrial weakening and impaired uterine integrity [10, 11].

The exact role of abnormal placentation in uterine rupture is unknown; however, it may result from myometrial weakening caused by the invasion of trophoblast into the myometrium, leading to thinning of the uterine wall and subsequent rupture.

In our case, the uterine rupture occurred in the second trimester. In most cases, uterine rupture occurs during the third trimester or labor, with an average of 36 weeks of pregnancy [12]. However, PAS has been reported to cause uterine rupture in early pregnancy, which complicates the diagnosis. A systematic review of 80 cases of second and early third‐trimester uterine rupture demonstrated PAS disorders in 33 cases (41.25%) [13]. Another systematic review reported eight cases of uterine rupture in the first trimester of pregnancy, and placenta percreta was confirmed in seven cases (87.5%) [14]. The rising incidence of CSs has led to an increase in the PAS prevalence. Thus, the life‐threatening complications of PAS must be considered in early and midgestational pregnancies.

In our case, the rupture occurred at the site of a previous cesarean scar. Uterine rupture can occur at either site of placenta protrusion or at the previous section scar. In late gestation, uterine rupture often involves dehiscence at the cesarean scar, whereas the uterine fundus is more commonly affected in first ruptures [15].

Our case was managed with hysterectomy. A retrospective analysis indicated that subtotal hysterectomy is the most commonly performed surgical intervention for uterine rupture, accounting for 73.6% of cases [16]. Previous studies have reported cases of coexisting uterine rupture and PAS that were managed conservatively [17]. Morken and Henriksen described a case of fundal uterine rupture in a young woman who underwent hysteroplasty and 2 years later had a normal pregnancy delivered by CS at 36 weeks [18].

At the 2‐year postpartum follow‐up, our patient experienced urinary urgency, as well as urge and stress incontinence. Consistent with this finding, a prior study of 84 women who underwent cesarean hysterectomy for PAS reported urinary urgency in 41 cases (48.8%) within 6–30 months postoperatively [19]. Additionally, overactive bladder has previously been reported following cesarean hysterectomy for uterine rupture associated with placenta percreta [20].

A limitation of this report is the lack of intraoperative images, which may reduce its value as a visual reference for surgical management in comparable cases. To mitigate this limitation, we summarized previously reported cases of uterine rupture due to placenta percreta during the second trimester in Tables 2 and 3, providing comparative clinical and surgical insights from the existing literature. However, what makes our case unique is the spontaneous bladder disruption following uterine rupture induced by placenta percreta.

**Table 2:** Maternal characteristics and clinical presentation in patients with uterine rupture due to placenta percreta in the second trimester.

	First author, year	Maternal age (years)	Obstetric history	Uterine operation history	GA (weeks)	Clinical presentation	Physical examination
1	Santoso, 2022 [20]	28	G2P1 (1CS)	Previous CS 2 years ago	24−25 weeks	Diffuse abdominal pain for 5 h with increased intensity, with no vaginal bleeding	Patient appeared very ill with weak pulse, diffuse rebound tenderness, tension pain on palpation of the cervix and uterine fundus was difficult to palpate, no vaginal bleeding, and protruding pouch of Douglas.
2	Dubbewa, 2022 [21]	29	G5P2L1A2 (2CS)	Two previous CSs 2 and 6 years ago and two surgical medical terminations of pregnancy via suction and evacuation	26 weeks	Sudden onset of generalized abdominal pain, vomiting, and retrosternal burning for 1 h, without vaginal bleeding or discharge.	Tachycardia (100/min), pallor, generalized abdominal tenderness with rebound tenderness, but no scar tenderness, closed cervix with no vaginal bleeding.
3	Bouab, 2022 [22]	36	G4P3 (3CS)	Three previous CSs	25 weeks	Acute abdominal pain for over 5 h, closed cervix, small amount of vaginal bleeding	Tachycardia (120/min), blood pressure difficult to measure, generalized mucocutaneous pallor closed cervix with minimal vaginal bleeding.
4	Saha, 2021 [23]	32	G2P1 (1CS)	CS 6 years ago, bilateral uterine artery ligation, B‐Lynch uterine compression sutures due to atonic postpartum hemorrhage	27 weeks + 2 days	Abdominal pain, fetal distress	Hemodynamic shock, gross pallor, cold peripheries, and distended and tense abdomen.
5	Omar, 2020 [24]	31	G5 P2Ab2 (2CS)	Two previous CSs, more than 5 years ago	20 weeks	Severe lower abdominal pain, vomiting	Unstable hemodynamic (tachycardia, hypotensive), soft abdomen, tender uterus (20‐week size), with no vaginal bleeding.

Abbreviations: CS, cesarean section; d, days; G, gravida; GA, gestational age; HR, heart rate; L, living; P, para; w, weeks.

**Table 3:** Surgical management and outcomes in patients with uterine rupture due to placenta percreta in the second trimester.

Case	Location of laceration	Management	EBL	Maternal outcome	Neonatal outcome
1	Left lateral uterine wall	Emergency exploratory laparotomy, hysterectomy	2 L	Full recovery discharged on Day 6, iatrogenic bladder trauma	Baby born alive, weighed 645 g, survived for 6 h
2	Lower uterine segment extending towards the fundus	Emergency exploratory laparotomy, hysterectomy, transfusion of 3 U blood	3 L of hemoperitoneum	Full recovery, discharged on Day 7	Death
3	Anterior uterine scar with placental protrusion	Emergency laparotomy, hysterectomy, transfusion of 7 U blood and 3 U FFP	2 L of hemoperitoneum	Full recovery	—
4	Fundus	Emergency laparotomy, hysterectomy, transfusion of 4 U packed RBC	3 L	Full recover discharged on Day 5	Death
5	Lower part of the anterior uterine wall	Emergency exploratory laparotomy, subtotal hysterectomy, transfusion of 7 U packed RBC	—	Full recovery discharge on Day 5	Death

Abbreviations: EBL, estimated blood loss; FFP, fresh frozen plasma; RBC, red blood cells; U, units.

In conclusion, this case report highlights the rare but life‐threatening potential of both uterine and bladder rupture associated with placenta percreta, even in the second trimester of pregnancy. Furthermore, when PAS is suspected antenatally, the possibility of uterine rupture should be considered throughout the pregnancy.

## Author Contributions

M.M.: conceptualization, investigation, and writing—original draft. L.P.: resources and supervision. S.B.: resources. L.S.: resources. A.V.: resources, supervision, project administration, and writing—review and editing.

## Funding

No funding was received for this manuscript.

## Disclosure

All authors have read and approved the final version of the manuscript. A.V. had full access to all of the data in this study and takes complete responsibility for the integrity of the data and the accuracy of the data analysis. M.M. affirms that this manuscript is an honest, accurate, and transparent account of the study being reported; that no important aspects of the study have been omitted; and that any discrepancies from the study as planned (and, if relevant, registered) have been explained. M.M. takes full responsibility for the content of the published article.

## Ethics Statement

This study was supported by the Mashhad University of Medical Sciences (Code: 4001397) and ethically approved by the Ethics Committee of the Mashhad University of Medical Sciences (Code: IR.MUMS.MEDICAL.REC.1401.043). The patient signed informed consent.

## Consent

Written informed consent was obtained from the patient for publication of this case.

## Conflicts of Interest

The authors declare no conflicts of interest.

## Data Availability

The data that support the findings of this study are available from the corresponding author upon reasonable request.
